# Hyponatremia: Prevalence and characteristics in internal medicine patients in southeast of China

**DOI:** 10.1097/MD.0000000000013389

**Published:** 2018-12-10

**Authors:** Xiaokun Gang, Yumin Zhang, Xin Pan, Weiying Guo, Zhuo Li, Yao Wang, Guixia Wang

**Affiliations:** a Department of Endocrinology and Metabolism, The First Hospital of Jilin University; b Department of Orthopedics, The Second Hospital of Jilin University, Jilin University, Changchun, China.

**Keywords:** electrolyte disturbances, epidemiology, hyponatremia, internal medicine

## Abstract

To determine the incidence, clinical features, etiology, risk factors, and mortality in internal medicine patients with hyponatremia (P-Na).

A prospective survey was conducted in patients with P-Na, diagnosed at admission in an Internal Medicine Department. 692 patients were then selected and subsequently divided into 3 groups based on the severity of P-Na. Multivariate linear regression analysis was used to explore the factors associated with levels of P-Na.

The prevalence of P-Na was 3.37%. Euvolemia P-Na was the predominant subtype in 3 types of P-Na (49.42%). Gastrointestinal and neurological manifestations were common hyponatremic symptoms. The leading 5 underlying diagnoses were chest infection (31.94%), malignancy (10.84%), cardiac disease (6.36%), liver cirrhosis (6.07%), and neurological disease (5.20%). Moderate and severe P-Na had higher mortalities than mild P-Na (*P* <.05). For the levels of serum Na, Age, and serum Cl were positively correlated while serum K, blood urea nitrogen (BUN), and Glu were negatively correlated (*P* <.05).

P-Na is common in internal medicine and accompanied by other electrolyte disturbances, various symptoms/diagnoses, and increased mortalities with decreasing Na, which requires special attention in clinical practice.

## 1 Introduction

Hyponatremia (P-Na), defined as a serum sodium (Na) concentration less than 135 mmol/L, is the most common electrolyte abnormality in current clinical practice. It is associated with various medical conditions (heart, liver, and kidney failure and the syndrome of inappropriate antidiuretic hormone (SIADH)), malignancies, and the use of specific medications.^[1]^

In most cases, P-Na is mild and asymptomatic, but sometimes it is associated with substantial gastrointestinal and neurological complications, especially when Na concentrations are below 120 mmol/L.^[1]^ P-Na is also associated with increased mortality and morbidity.^[2]^ It is uncertain however as to whether patients with P-Na tend to be elderly and whether the mortality risk is related to the degree of P-Na.^[3,4]^

Previous studies have described hyponatremic patients in nursing homes, hospital wards, intensive care units, emergency departments, ambulatory clinics and cancer centers in different countries.^[5–8]^ There are few reports of P-Na, within small sample sizes, in an internal medicine setting in northeastern China. The aim of our study was to investigate the frequency, clinical and biochemical features, underlying diagnoses, and clinical outcomes in hyponatremic patients presenting to the department of internal medicine. We closely documented presenting symptoms of hyponatremic patients to ensure clinicians were apprised of their clinical conditions.

## 2 Material and methods

### 2.1 Subjects

The present study is a descriptive, retrospective hospital record analysis. We searched the laboratory database of the general internal medicine department of the local Hospital to identify all patients with P-Na (P-Na-value <135 mmol/L). We excluded inpatients from the departments of surgery and emergency, and minors (less than 18 years of age) from any department. Only first visits were registered for those with multiple visits.

P-Na was analyzed in all patients in the internal medicine department. In patients with diagnosed hyperglycemia or hyperlipidemia, P-Na-values were corrected before they were enrolled.^[9]^ If the corrected P-Na-value returned to the normal range, then we attributed the low value to pseudo-P-Na and excluded the patient. If the corrected P-Na-value was still below the normal range, we concluded that there might be other factors leading to P-Na.

Among 20,534 total patients from 18 to 90 years of age between November 2011 and April 2012, 692 patients (60.26% male) were identified as hyponatremic. We classified the patients according to the clinical severity of P-Na as mild (group 1: P-Na 130–134 mmol/L), moderate (group 2: P-Na 120–129 mmol/L), and severe (group 3: P-Na < 120 mmol/L).^[1,9]^ The records of these patients were reviewed for relevant demographic, clinical, and laboratory data, in addition to underlying diagnoses, causes, and outcomes of hospitalization. Serum electrolytes were measured by an ion-selective electrode system on an ACCESS chemiluminescence analyzer (intrabatch coefficients of variation were <1.5% and <4.0%). Informed consent was signed by all patients. The study protocol was reviewed and approved by the Ethics Committee of the First Hospital of Jilin University.

### 2.2 Statistical analysis

Results from univariate analyses were expressed as mean ± standard deviation (mean ± SD). Independent sample t-tests and chi-square tests were used for bivariate analyses. Analysis of variance (ANOVA) was used for comparison involving more than 2 groups. Multivariate linear regression analyses were used to explore the possible factors associated with Na level. *P* <.05 was considered statistically significant. Data were analyzed using SPSS 17.0 statistical software.

### 2.3 Reference values

Serum Na: 135 to 145 mmol/L; serum potassium (K): 3.5 to 5.5 mmol/L; serum calcium (Ca): 2.2 to 2.6 mmol/L; serum chloride (Cl): 98 to 108 mmol/L; glucose (Glu): 3.9 to 6.1 mmol/L; blood urea nitrogen (BUN): 3.2 to 7.0mmol/L; creatinine (CRE): 44 to 115 μmol/L; albumin (ALB): 35 to 55 g/L.

## 3 Results

### 3.1 Patient characteristics

The median age of the 692 patients with P-Na was 57.12 ± 16.68 years. Baseline characteristics of the patients were recorded according to the severity of P-Na (Table 1). No significant differences were found in age, sex, systolic blood pressure (SBP), diastolic blood pressure (DBP), serum K, CRE, or Glu. Serum Ca was significantly lower in patients with moderate (*P* <.01) and severe (*P* <.05) P-Na than in the mild group. BUN (*P* <.01) was significantly higher and ALB was significantly lower in the moderate group than in the mild group, although there were no significant differences in these values between the mild and severe groups. We further estimated their body fluid status as previous literature reported.^[10]^ According to the hyponatremic patients’ clinical manifestations (e.g., blood pressure, pulse, urine output, skin elasticity, color, and temperature changes), we classified them into 3 subtypes (hypovolemic, euvolemic, and hypervolemic). The most common body fluid subtype in each group was euvolemic P-Na, but there were no significant differences between the subtypes (Table 1).

**Table 1:**
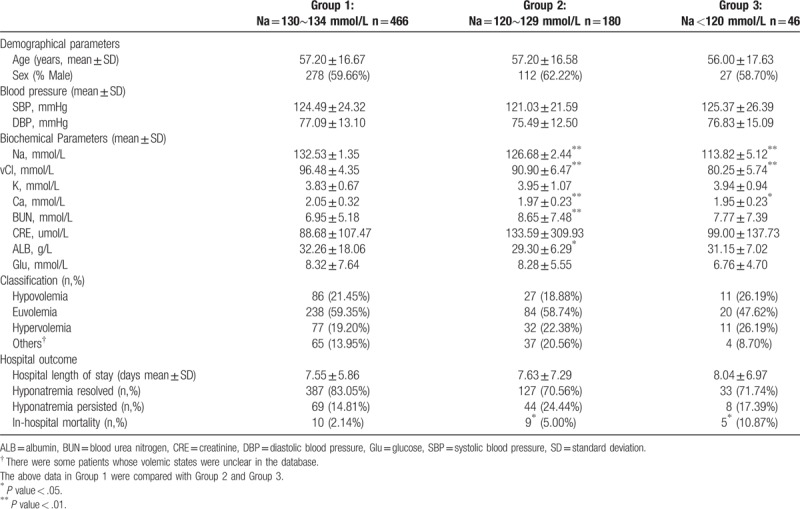
Baseline characteristics of the subjects according to severity of P-Na.

We utilized the clinical data to perform multivariate linear regression (Table 2). Age and serum Cl were positively correlated with serum Na, while serum K, BUN, and Glu were negatively correlated with serum Na.

**Table 2:**
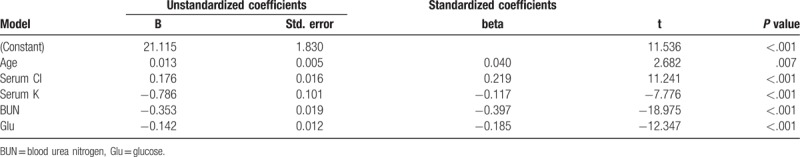
Indicators correlated with P-Na.

### 3.2 Symptoms of P-Na and clinical diagnoses

Patients reported gastrointestinal, neurological, and muscular symptoms that may have been caused by P-Na (Table 3). Each P-Na severity group had 2 concomitant symptoms while only 1 patient with moderate P-Na had 3 concomitant symptoms.

**Table 3:**
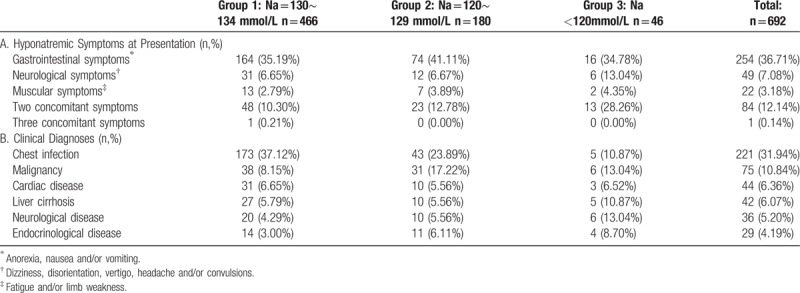
Hyponatremic symptoms and clinical diagnosis.

The most common diagnoses associated with P-Na were chest infections, malignancies, cardiac disease, liver cirrhosis, and neurological disease (Table 3). Of the 29 hyponatremic patients with endocrinological diseases, 19 were due to hyperglycemia (true P-Na was established after correction for hyperglycemia as previously described). Hypopituitarism (4 cases), hypothyroidism (2 cases), Addison's disease (2 cases), and hyperlipidemia (2 cases) were more often seen in moderate and severe P-Na patients.

We considered the diagnosis of SIADH based upon 5 criteria:

1) P-Na, serum Na < l35mmol/L; 2. urine osmolality in excess of plasma osmolality
2) (<280 mOsm/kg·H_2_O);
3) natriuresis >20 mmol/d;
4) absence of edema and volume depletion;
5) normal heart, kidney, liver, adrenal gland, and thyroid function).^[11]^
6) However, as urine osmolality was not performed on all our patients, we used the urine specific gravity to estimate the urine osmolality in some patients (urine specific gravity were performed on all patients).^[10]^ Among the patients with euvolemic hypernatremia, 336 met the criteria for SIADH.

The dominant underlying etiologies were pulmonary disorders (n = 230, 68.45%), malignancies associated with SIADH (n = 69, 20.54%) and neurological diseases (n = 37, 11.01%). We collected the drug histories of all patients; however, we did not identify any medication that correlated with SIADH.

### 3.3 Hospital outcome

The length of hospital stay was not significantly different between mild, moderate, and severe P-Na groups (Table 1). Before discharge, P-Na resolved in 547 (79.05%) patients and persisted in 121 (17.49%) patients. The in-hospital mortality of the population was 2.14%, 5.00%, and 10.87% in the mild, moderate, and severe P-Na groups, respectively. Mortality was significantly different between the groups (*P* <.05).

## 4 Discussion

In recent years, there have been efforts to perform epidemiological studies on P-Na in different subgroup hospitalized patients, but few studies about the overall characteristics and analysis were reported from patients in Northeast China. Knowing the clinical factors and outcomes associated with P-Na and establishing common underlying causes can be very useful in early disease prevention and raising more attention from the doctors in this area.

In this descriptive study, the prevalence of P-Na (P-Na <135 mmol/L) in internal medicine was 3.37% which was among the previously reported incidence of P-Na ranging from 3 to 40% depending upon the definitions of the disturbance and the population surveyed.^[12–15]^ Sex differences in the incidence of P-Na have been noted previously. Wilkinson et al found females to be at significantly greater risk of P-Na when coupled with low body weight.^[16]^ In another study that included a mixed Asian sample, gender was not correlated with disturbances in serum Na concentration.^[17]^ The gender difference could be attributable to ethnic differences in body composition as Asians have higher body fat levels than Caucasians.^[18]^ We also found no significant correlation between gender and P-Na in our study, which had more male participants in each subgroup. Since our study included only Chinese participants from the northeast, our results may not be generalizable to other populations.

Previous epidemiological studies have shown an increased prevalence of P-Na in the elderly.^[7,19]^ In our study, age was positively correlated with serum Na, meaning that younger patients had lower levels of serum Na. The relatively low average age of our study sample (57.12 ± 16.68 years) could have contributed to this result. We also found serum K, BUN, and Glu to be negatively correlated with serum Na, although positively correlated with serum Cl. As with the decreasing P-Na, electrolytic disturbances such as hypochloremia, hyperglycemia, and hyperkalemia can be more common.

No clear underlying cause has been found in the 13% to 23% of patients with milder P-Na. Patients had gastrointestinal, neurological, and muscular symptoms and clinical diagnoses varied from chest infections to endocrine diseases in the present study. Most of the patients in our study had clinical euvolemia, which was likely partially attributable to the large number of patients (n = 336, 48.55%) who met criteria for SIADH with the highest proportion being pulmonary disorders, followed by malignancies and central nervous system disorders. Our results for SIADH were similar to previous studies.^[20,21]^ However, our results must be interpreted with caution since we did not have all the data necessary to definitively diagnose SIADH.

In our study, we found that mortality of moderate and severe P-Na was greater than that of mild P-Na. These results were in agreement with studies done in intensive care units.^[22]^ However, a large hospital-based study found that serum Na levels correlated with the severity of underlying diseases that were the likely causes of the deaths.^[23,24]^

Our study was observational, so we cannot draw firm conclusions regarding causality between suggested aetiologies and P-Na. We were not able to test our hypotheses regarding how sex and age correlate with P-Na.

P-Na was often accompanied by other electrolyte disturbances such as hypochloremia, hyperglycemia, and hyperkalemia. Chest infection, malignancy, cardiac disease, liver cirrhosis, and neurological disease were the most common underlying diagnoses of P-Na in our retrospective study. Gastrointestinal and neurological manifestations were common hyponatremic symptoms at presentation. The severity of P-Na was associated with in-hospital mortality.

## 5 Conclusion

We found P-Na to be a common condition among patients presenting for care in an internal medicine department in a northeastern Chinese hospital. P-Na was often accompanied by other electrolyte disturbances such as hypochloremia, hyperglycemia, and hyperkalemia. The severity of P-Na was associated with in-hospital mortality. The symptoms of P-Na and the diagnoses that co-occur with the condition vary widely, requiring individualized treatment regimens.

## Author contributions

**Data curation:** Weiying Guo.

**Formal analysis:** Yumin Zhang, Guixia Wang.

**Funding acquisition:** Guixia Wang.

**Investigation:** Xin Pan.

**Methodology:** Guixia Wang.

**Resources:** Guixia Wang.

**Validation:** Zhuo Li.

**Writing – original draft:** Yao Wang.

**Writing – review & editing:** Xiaokun Gang.
