# Hyponatremia: Prevalence and characteristics in internal medicine patients in southeast of China: Erratum

**DOI:** 10.1097/MD.0000000000014243

**Published:** 2019-01-18

In the article, “Hyponatremia: Prevalence and characteristics in internal medicine patients in southeast of China”,^[1]^ which appeared in Volume 97, Issue 49 of *Medicine*, the title should be “Hyponatremia: Prevalence and characteristics in internal medicine patients in northeast of China.”
